# Treatment of Immature Teratoma Associated with Ovarian Endometrioma: A Case Report

**DOI:** 10.18502/jri.v24i3.13278

**Published:** 2023

**Authors:** Soheila Amini Moghaddam, Niloufar Sarchami, Ali Rahbari

**Affiliations:** 1- Department of Obstetrics and Gynecology, Firouzgar Hospital, Iran University of Medical Sciences, Tehran, Iran; 2- Department of Pathology, Jam Hospital, Tehran, Iran

**Keywords:** Endometrioma, Fertility preservation, Immature teratoma, Neoadjuvant chemotherapy

## Abstract

**Background:**

Mature teratoma is a benign neoplasm, mostly composed of well-differentiated derivations of almost two or three germ cell layers, while immature teratoma is a malignant neoplasm composed of immature neural and embryonic tissue. Immature teratoma in the context of ovarian endometrioma has not been reported yet.

**Case Presentation:**

A 34-year-old woman with primary infertility is reported in this study who suffered from immature teratoma associated with ovarian endometrioma. After several rounds of fertility treatment, the patient was referred for severe abdominal pain and underwent emergency surgery for the rupture of ovarian endometrioma. To preserve the ovary, the cyst was not resected in areas attached to the ovary. Some months later, the patient noticed a progressive abdominal enlargement. The sonographic evaluation revealed multiple solid-cystic lobulated masses on the abdominal wall and throughout the pelvic cavity. The histologic diagnosis was consistent with immature teratoma. The patient was treated with high-dose neoadjuvant chemotherapy and fertility-sparing surgery (FSS). The histologic evaluation of the extracted masses revealed teratoma maturation.

**Conclusion:**

This study reveals the importance of complete removal of endometrioma and highlights the role of neoadjuvant chemotherapy in fertility-sparing surgery and potentiating teratoma maturation.

## Introduction

Endometriomas are benign and common ovarian cysts that are found in 15–42% of females suffering from endometriosis (1). Mature cystic teratomas are also common cysts of the female reproductive system, accounting for 10–20% of all ovarian cysts (1). The coexistence of endometrioma and mature cystic teratomas has been rarely reported and is regarded as a diagnostic and therapeutic challenge (1). However, endometrioma in association with immature teratoma has not been reported in earlier studies.

Mature teratoma is a benign neoplasm, mostly composed of well-differentiated derivations of almost two or three germ cell layers, while immature teratoma is a malignant neoplasm composed of immature neural and embryonic tissue (2). The management of advanced-stage ovarian immature teratoma is challenging as it requires high-dose chemotherapy with serious potential chemotherapy-induced complications. In addition, immature teratomas may be resistant to standard chemotherapy regimens (3, 4).

In this report, a complicated case of a 34-year-old woman is reported who suffered from endometrioma associated with immature teratoma that was managed with neoadjuvant therapy followed by fertility-sparing surgery.

## Case Presentation

This case was presented in March 2022 in Firouzgar Hospital of Iran University of Medical Sciences. A 34-year-old female nurse, with a history of dyspareunia and dysmenorrhea and a diagnosis of primary infertility, was referred to a gynecologist for the treatment of her symptoms. The patient had normal FSH (6.3 *mIU/ml*), LH (11.1 *mIU/ml*), HE4 (63.2 *ng/ml*), and AMH (5.13 *ng ml*), and elevated CA-125 (371.1 *U/ml*) and ROMA-index (14.6%). The patient underwent hysterosalpingography to detect the cause of infertility, which was normal with no blockage in the fallopian tube and uterus. The patient received several courses of letrozole that were not effective in the treatment of infertility.

Almost five months later, the patient was referred to the emergency department of a teaching hospital with severe abdominal pain. The sonographic evaluation revealed a very large cystic lesion (74*95*108 *mm*) with volume of 400 *ml* in the midline position of the pelvic, raising the probability of ovarian torsion. The amount of free fluid around the cyst was about 85 *ml*. The patient underwent an emergent operation for the rupture of ovarian endometrioma which spread throughout the abdominal cavity. The surgery was done by a general gynecologist. In order to preserve the ovary for future ovulation and pregnancy, the surgeon did not resect the cyst in areas firmly attached to the ovary. After the left ovarian cystectomy, an abdominal drain was placed and the patient was discharged afterward. The histologic examination of the tissue specimen from the cyst wall was consistent with the diagnosis of endometrioma.

Almost two months later, the patient was referred to an obstetrician and gynecologist for an infertility problem. Before ovulation induction, a sonographic evaluation was done, which showed an endometrioma in the left ovary (40 *mm*) with an echogenic center of 25 *mm*, without internal blood flow. Furthermore, moderate ovarian attachment to the uterus and bilateral uterosacral ligament thickening were noticed. The patient received high-dose of medication for ovulation induction (Cinnal-F, 24 units) for 12 days. From the first days of medication, the patient felt the presence of a progressively growing palpable cyst in the exact site of the previous abdominal drain. However, the doctor assured her that they are endometriosis nodules in the abdominal wall and are nothing to be concerned about. She only performed follow-up vaginal sonographic evaluations which showed the adequate size and number of follicles in both ovaries. Therefore, the ovarian puncture was performed as planned. However, no abdominal sonographic evaluation was performed to investigate the abdominal wall nodules.

Almost three weeks after the ovarian puncture, the patient was referred again to the emergency department or hospital with severe abdominal pain and bleeding. The sonographic evaluation revealed two cysts with sizes of 46*61 *mm* and 37*53 *mm* with intermediate echo in the left ovary which was in favor of endometrioma. Slight amounts of free pelvic fluid were also observed. Concurrent sonographic evaluation of soft tissues of abdominal wall demonstrated two hypo-heteroechoic masses with the sizes of 22*35 *mm* and 79*99 *mm* on the right side of the surgical scar in the hypogastric region, with the diagnoses in favor of endometriosis nodules.

**Figure 1.**
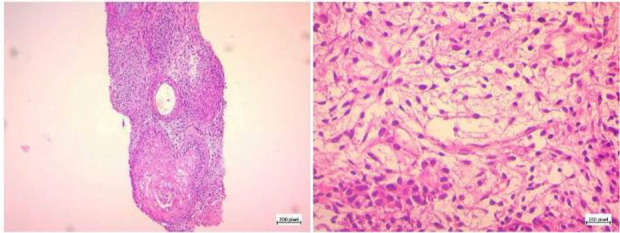
Histologic examination before second-look surgery consistent with the diagnosis of immature teratoma

In the following two months, the patient’s abdomen showed a progressive enlargement, so the patient required clothing two sizes larger. Again, the patient visited her own infertility doctor and sonographic evaluation revealed a large cyst of the abdominal wall with the approximate size of 10 *cm*. Sonographic evaluations demonstrated multiple solid-cystic lobulated masses located on the abdominal wall and throughout the pelvic cavity indicating malignancy (sarcomatosis). The patient underwent a biopsy. Histologic evaluation of the biopsy sample showed immature neuroglial tissue and neuroepithelial rosettes in the context of cellular stroma, consistent with the diagnosis of immature teratoma (Figure 1).

**Figure 2.**
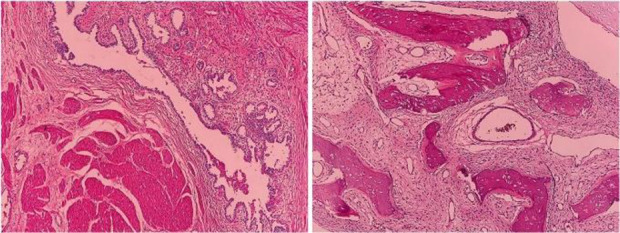
Histologic examination after the second-look surgery consistent with the diagnosis of mature teratoma

The patient received four courses of chemotherapy with BEP (Bleomycin + Etoposide + Cisplatin) regimen. The chemotherapy of the patient was associated with numerous complications including chemotherapy-induced neuropathies and several infections leading to hospitalization. Three months after chemotherapy, the patient was elected for surgical debulking of tumors of the abdominal wall and pelvic cavity by a gynecologist and a surgical oncologist. Due to the wide adhesion of the tumor to the abdominal wall, a substantial part of the abdominal wall was resected. The debulking of multiple huge abdominopelvic tumors of about 10*20 *mm* was done completely. In addition, the left salpingo-oophorectomy was done. A large section of the sigmoid was damaged intraoperatively, which led to Hartmann’s procedure and colostomy. The extracted masses were sent to the pathology department for histologic examination, which showed mature glial tissue and neurons and a well-differentiated cerebellar tissue, consistent with the diagnosis of mature teratoma (Figure 2). Two days after the debulking surgery, the patient underwent the second surgery for abdominal wall reconstruction with mesh.

After the operation, the patient had some difficulties doing her routine activities such as walking, which required several occupational therapy sessions. During the three months of follow-up, the patient required two further surgeries for the repair of the implanted meshes in the abdominal wall. No sign of recurrence was observed until the last follow-up.

## Discussion

Malignant germ cell tumors are rare and mostly involve females in their reproductive years. Surgery is the first and most important option in the treatment of malignant germ cell tumors. Fertility-sparing surgery that allows preserving one ovary and the uterus has shown long-term safety for young women seeking future pregnancy and is also effective in preventing early menopause. However, recent evidence suggests avoiding FSS in cases with an increased risk of peritoneal adhesions (5).

Immature ovarian teratoma is a rare germ cell tumor with challenging diagnosis and treatment (6). Its concurrence with other lesions makes its diagnosis and treatment even more complicated. The concurrence of mature ovarian teratoma and endometrioma has been reported in some earlier studies. Matalliotakis et al. reported the coexistence of mature ovarian teratoma with endometrioma in 2.9% (7) of cases. To the best of our knowledge, immature ovarian teratoma in association with endometrioma has not been reported in earlier literature. By the present report, the coexistence of immature teratoma and endometrioma can not be confirmed in our case, because the first histologic examination was consistent with the diagnosis of endometrioma. The most probable scenario is the malignant transformation of endometriosis or the remnants into an immature teratoma (8). If this is the case, complete removal of the cyst in the index surgery by a more specialized surgeon (gynecologic oncologist) could be a more valuable option than preserving the ovary for future ovulation. In that case, the significant adverse effects of the chemotherapy and surgical complications could be avoided and treatment costs could be significantly reduced.

While the role of extensive surgery is acknowledged for the treatment of ovarian germ cell tumors, there is no consensus on the timing of chemotherapy (9). Adjuvant chemotherapy is reported as the standard choice of treatment for adult women with advanced-stage immature teratoma (10). However, in a number of cases, neoadjuvant chemotherapy is implemented for the treatment of advanced ovarian germ cell tumors to reduce the tumor bulk and to increase the feasibility of fertility-sparing surgery (11). Neoadjuvant chemotherapy allows a more precise anatomic definition of the target mass volume. In addition, histological modification of an immature teratoma to a mature teratoma during or after neoadjuvant chemotherapy, termed growing teratoma syndrome, might occur in a number of cases (12). However, there is always a risk of losing the opportunity for debulking surgery due to significant side effects of neoadjuvant chemotherapy. Therefore, an appropriate selection of patients is necessary when opting for neoadjuvant chemotherapy (13). In the present case, neoadjuvant therapy was selected to reduce the tumor bulk and allow performing FSS. Although significant side effects of chemotherapy occurred in our patients, the FSS was successful, so one ovary was preserved for fertility. However, the follow-up period of the study was not long enough to confirm the success of surgery in tumor control and prevention of recurrence. Second-look surgery could be implemented in patients with persistent radiologic abnormalities (14).

## Conclusion

Altogether, the present report reveals that complete removal of endometrioma overweighs preserving fertility because mismanagement of endometrioma could be associated with subsequent devastating outcomes. In addition, the present case highlights the role of neoadjuvant chemotherapy in the management of ovarian immature teratoma, by increasing the feasibility of fertility-sparing surgery and the possibility of teratoma maturation.
